# Overcoming the Damping–Elasticity Paradox via 3D‐Printed NiTiSn Nanocomposite

**DOI:** 10.1002/advs.202506410

**Published:** 2025-06-10

**Authors:** Bo Feng, Helong Liu, Hui Shen, Ying Yang, Fangmin Guo, Lishan Cui, Yang Ren, Jie Chen, Shuke Huang, Yao Xiao, Zhihui Zhang, Hongxiang Zong, Yinong Liu, Shijie Hao

**Affiliations:** ^1^ College of New Energy and Materials China University of Petroleum Beijing 102249 China; ^2^ College of Materials Science and Engineering Hohai University Changzhou 213200 China; ^3^ College of Chemical Engineering and Environment China University of Petroleum Beijing 102249 China; ^4^ Department of Physics City University of Hong Kong Hong Kong 999077 China; ^5^ Institute of Machinery Manufacturing Technology China Academy of Engineering Physics Mianyang 621900 China; ^6^ School of Mechanical Engineering Tongji University Shanghai 201804 China; ^7^ State Key Laboratory of Bionic Engineering (Ministry of Education) Jilin University Changchun 130025 China; ^8^ State Key Laboratory for Mechanical Behavior of Materials Xi'an Jiaotong University Xi'an 710049 China; ^9^ Department of Mechanical Engineering The University of Western Australia Perth 6009 Australia

**Keywords:** 3D printing, damping‐elasticity paradox, nanocomposites, reversible detwinning‐twinning, shape memory alloys

## Abstract

Developing high damping alloys (HDAs) with large elastic strain has attracted growing attention due to the increasing demand for energy absorption with overload reliability and reusability. However, damping capacity inherently conflicts with elasticity, because the former requires a liable movement of crystal defects while the latter opposite. To deal with the damping‐elasticity paradox, the advantage of pseudobinary eutectic reaction and rapid cooling of laser powder bed fusion is taken to fabricate a bulk NiTiSn nanocomposite with a two‐level hierarchical structure. The first‐level architecture is composed of martensitic NiTi nanolamellae and reinforced Ti_3_Sn nanolamellae. In addition to lattice strain matching and lamellar boundary strengthening, a novel mechanism of martensite reorientation mediated by reversible stress‐induced detwinning‐twinning is activated to generate large elastic strain. A high density of nanotwins and nanodomains within NiTi nanolamellae constitute the second‐level architecture, which provides pronounced internal friction for high damping capacity. As a result, our NiTiSn nanocomposite exhibits a record‐high integration of damping capacity (tanδ > 0.10) and elastic strain (exceeding 4.5%), as well as superb stability under cyclic overload. This research not only represents a major breakthrough in achieving HDAs with outstanding damping and elastic strain but also offers a novel paradigm for high‐performance functional and structural materials.

## Graphical abstract

Here a 3D‐printed nanoeutectic alloy is designed to exhibit a record‐breaking combination of exceptional damping capacity (tanδ > 0.10) and elastic strain exceeding 4.5% at a high stress ≈1500 MPa, overcoming the damping‐elasticity paradox in high damping alloys.

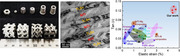

## 1 Introduction

The substantial demand for vibration control in optical instruments, automobiles, buildings, bridges, and aerospace devices has driven the rapid development of high‐damping alloys (HDAs).^[^ ^1^, ^2^, ^3^, ^4^ ^]^ Overloading, although occasional in these scenarios, is almost inevitable over long‐term usage. ^[^ ^5^, ^6^ ^]^ Therefore, besides energy absorption, elastic strain limit is a critical metric for emerging sustainable HDAs.^[^ ^7^, ^8^ ^]^ However, a prevailing issue is that the damping capacity of alloys, which is positively related to the density and mobility of crystal defects,^[^ ^4^, ^9^, ^10^, ^11^, ^12^, ^13^ ^]^ inherently conflicts with large elastic strain, which requires the suppression of crystal defect generation and movement.^[^ ^14^, ^15^ ^]^ To date, developing HDAs with large elastic strain remains an insurmountable challenge.

Among vast material systems, NiTi alloy in the B19’ martensite phase (B19’‐NiTi) is known as a promising HDA due to abundant movable twinning interfaces among and within variants.^[^ ^16^, ^17^, ^18^ ^]^ Like other HDAs, B19’‐NiTi exhibits a small elastic strain less than 0.5%.^[^ ^19^, ^20^ ^]^ However, we found that the nano‐reinforced phase embedded in the B19’‐NiTi matrix manifests an ultra‐large elastic strain of several percent via a mechanism known as lattice strain matching.^[^ ^21^, ^22^, ^23^ ^]^ Based on this finding, we hypothesize that a nanocomposite composed of nanoscale B19’‐NiTi phase and nano‐reinforced phase may be a feasible solution to the damping‐elasticity paradox. During loading, nanoscale B19’‐NiTi gradually launches detwinning under high stress. Lattice strain matching postpones the yielding of the nano‐reinforced phase and endows it with an ultra‐large elastic strain. In this process, the strong constraint from the nanoscale boundaries causes the accumulation of substantial mechanical elastic energy in both phases. After the external load is removed, the elastic energy stored upon loading is converted into the driving force to stimulate the re‐twinning of nanoscale B19'‐NiTi. The resultant recovery of shape and microstructure improves the overall elastic strain and restores the intrinsic damping source of the nanocomposite.

The aforementioned material design scheme relies heavily on the size refinement and interfacial compatibility between NiTi and reinforced constituents, accordingly, in situ composites with hetero‐alternating lamellae formed through NiTi‐X pseudobinary eutectic reaction are competent candidates. For the purpose of improving lattice strain matching, nanoeutectic alloy is preferred but conventional metallurgical methods can only produce eutectics with sub‐micron or micron lamellae.^[^ ^24^, ^25^, ^26^ ^]^ As a typical 3D printing technique renowned for its precise in manufacturing metallic parts with complex geometries, laser powder bed fusion (LPBF) is characterized by a rapid local non‐equilibrium solidification at a cooling rate exceeding 10^5 ^°C s^−1^, which is four orders of magnitude greater than conventional casting.^[^ ^27^, ^28^, ^29^ ^]^ Since the thickness of eutectic lamellae is inversely proportional to the cooling rate,^[^ ^30^, ^31^ ^]^ we expect that the NiTi‐based eutectic‐type nanocomposite produced by LPBF will exhibit a markedly refined microstructure and distinct mechanical response compared to its counterpart produced by conventional metallurgical methods.

In this study, a NiTi‐Ti_3_Sn eutectic‐type nanocomposite with a two‐level hierarchical structure was produced by LPBF. The first‐level architecture is composed of alternating NiTi nanolamellae (as fine as 57 nm) and Ti_3_Sn nanolamellae (as fine as 40 nm). A novel martensite reorientation mechanism mediated by reversible stress‐induced detwinning‐twinning, in conjunction with lattice strain matching and enhanced lamellar boundary strengthening, contributes to a large elastic strain over 4.5%. The second‐level architecture is made up of highly mobile (001)_B19’_ compound nanotwins and R nanodomains in NiTi nanolamellae, endowing the overall material with an exceptional damping capacity (tanδ > 0.10). These unique microstructures, unprecedented mechanisms and record‐breaking metrics reflect a major breakthrough in achieving HDAs with combined superb damping and large elastic strain.

## 2 Results

### 2.1 Hierarchical Structure of Nanocomposite

**Figure 1:**
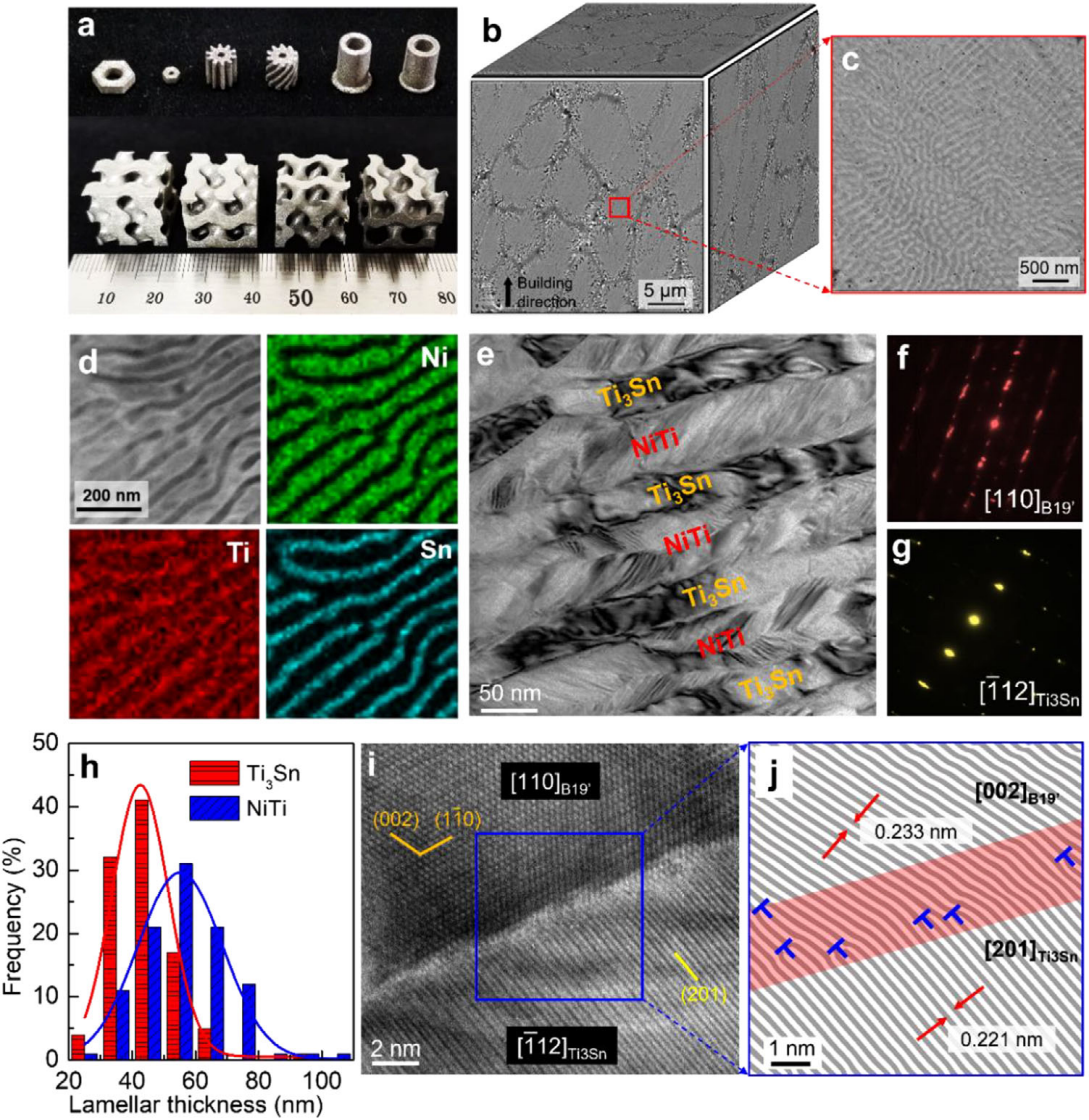
a) 3D‐printed NiTiSn components including fasteners, gears, and periodically porous structures; b) 3D backscattered scanning electron microscope (SEM) image of the as‐printed NiTiSn showing nearly equiaxed cellular structures; c) Backscattered SEM image of nanolamellae within a cellular structure; d) Scanning transmission electron microscopy energy dispersive X‐ray (STEM‐EDX) elemental maps of nanolamellae; e) Bright‐field TEM image of alternating NiTi and Ti_3_Sn nanolamellae; f,g) Selected area electron diffraction (SAED) patterns with zone axes of [110]_B19’_ (showing that the NiTi component is indexed as B19’ phase) and [$\bar{1}$12]_Ti3Sn_, respectively; h) Thickness distributions of nanolamellae in the as‐printed NiTiSn; i) High resolution TEM (HRTEM) image showing the NiTi/Ti_3_Sn interface of the as‐printed NiTiSn; j) Local inverse fast Fourier transform (IFFT) image from (i) showing the interplanar spacings of [002]_B19’_ and [201]_Ti3Sn_ are 0.233 and 0.221 nm, respectively. The interface between B19’‐NiTi and Ti_3_Sn is highlighted in red. Interfacial dislocations are identified and marked with blue “T” symbols.

We applied LPBF to melt and deposit pre‐alloyed Ni_34_Ti_58_Sn_8_ (at.%, pseudobinary NiTi‐Sn eutectic composition,^[^ ^32^ ^]^ Figure S1, Supporting Information) powders in a layer‐by‐layer manner (laser scanning strategy is shown in Figure S2a, Supporting Information). LPBF parameters were searched for high relative density (> 99%) and good forming quality (Figures S2b,c and S3, Supporting Information). Provided the optimized LPBF parameters (laser power 50 W, hatch spacing 20 µm and scanning speed 150 mm/s, Figure S2b, Supporting Information), we fabricated various 3D‐printed components with complex geometries, including fasteners, gears and periodically porous structures (**Figure** **1**a), demonstrating high geometric designable freedom of bulk NiTiSn nanocomposite rendered by LPBF. For an in‐depth discussion, we chose as‐cast Ni_34_Ti_58_Sn_8_ (at.%) as a reference material.

As shown in Figure 1b–d and Figure S4a,b (Supporting Information), near‐equiaxed cellular structures filled with nanolamellae are generated by LPBF. Close‐up TEM characterization shows that the as‐printed NiTiSn consists of alternating martensitic NiTi (B19’ type) and Ti_3_Sn nanolamellae (Figure 1e–g; Figure S4c, Supporting Information) with average thicknesses of 57 and 40 nm (Figure 1h), respectively. These nanolamellar colonies with random orientations enable the as‐printed bulk material an overall microstructural isotropy, which is different from the strong anisotropy in nanolayered alloys fabricated through thin‐film deposition and severe plastic deformation.^[^ ^30^ ^]^ The volume fraction of the Ti_3_Sn phase is ≈41% by image analysis. Since the lamellar thickness of the as‐cast sample with the same ingredient approaches the micrometer scale (Figure S5, Supporting Information), it is verified unambiguously that the strikingly strong eutectic refinement in the as‐printed sample is achieved by the rapid cooling rate of LPBF (>10^5^ K/s).^[^ ^28^, ^30^ ^]^ An enlarged view of the region adjacent to the nanolamellar boundary is presented in Figure 1i. The corresponding IFFT map (Figure 1j) shows that the misorientation and the lattice misfit between [002]_B19’_ and [201]_Ti3Sn_ are 15° and 5.43%, respectively. This semi‐coherent interface is accommodated by profuse interfacial dislocations.

**Figure 2:**
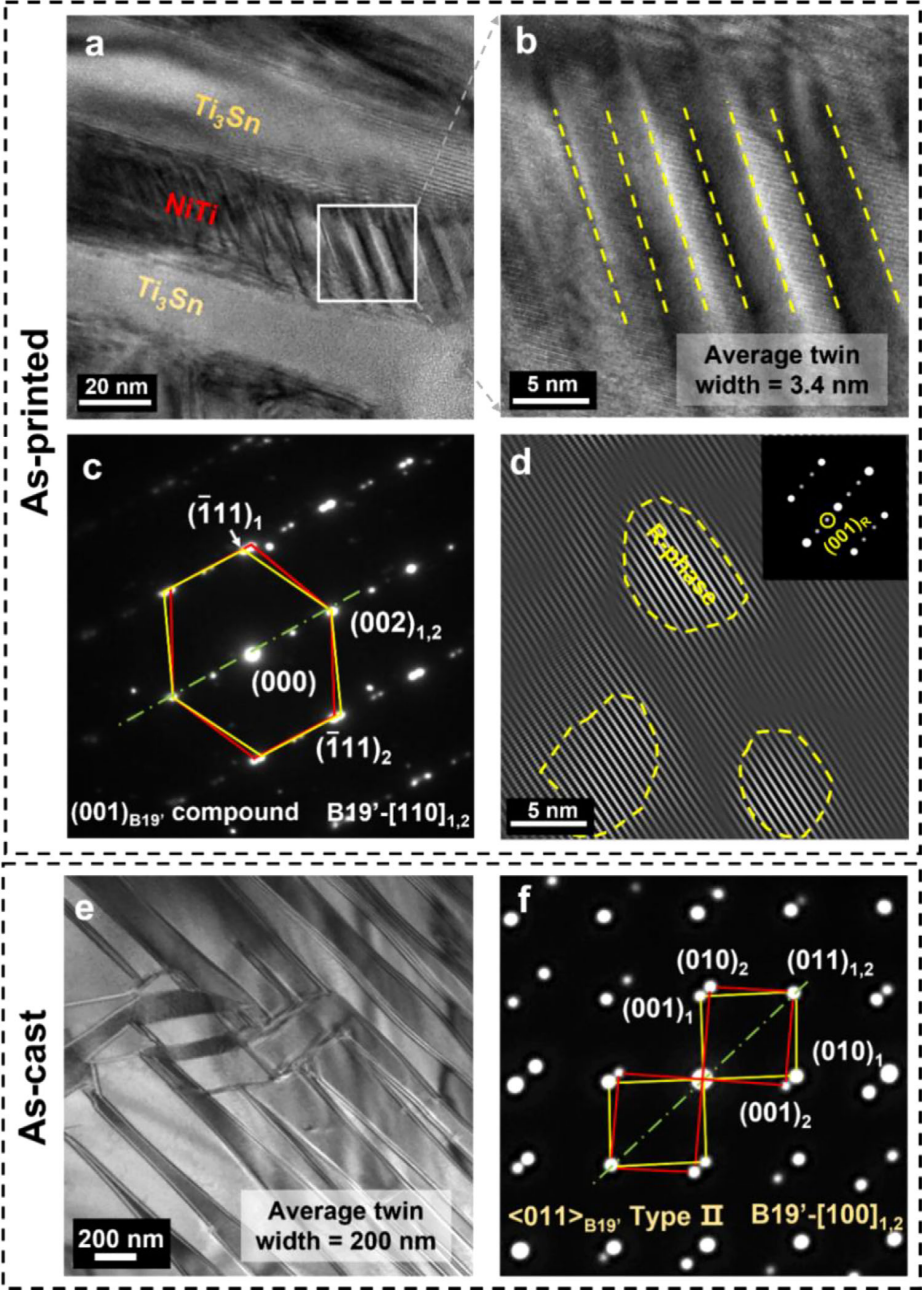
a) HRTEM image of nanolamellae in the as‐printed NiTiSn. NiTi nanolamellae are twinned while Ti_3_Sn nanolamellae are not; b) Magnification of the white box in (a) revealing martensite nanotwins. Twinning boundaries are marked by yellow dash lines; c) SAED pattern of (b) showing (001)_B19’_ compound twins; d) IFFT image of NiTi near the interface showing some R‐phase nanodomains visualized by (001)_R_ reflection (yellow solid circle in the inset). Domain boundaries are marked by yellow dash circles; e) Twinning morphology of B19’ martensite in the as‐cast NiTiSn; f) SAED pattern of (e) showing <011>_B19’_ type II twins.

**Figure** **2**a–c elucidates that nanolammellar NiTi phase in the as‐printed NiTiSn is primarily composed of (001)_B19’_ compound nanotwins with an average width of 3.4 nm. It is worth noting that (001)_B19’_ compound twins normally exist in nanograins or in thin films that contain high‐density precipitates.^[^ ^33^, ^34^, ^35^ ^]^ Here we verify that (001)_B19’_ compound twins can also be activated by the intense geometrical constraints from nanoeutectics. In some regions near the nanolamellar interface, the intermediate martensite, namely R‐phase nanodomains (Figure 2d; Figure S4d, Supporting Information) with various sizes of 3–10 nm, prevails over B19’ nanotwins because R‐phase is prone to be stable under the drastically fluctuated strain field created by nanostructures.^[^ ^36^ ^]^

With respect to the as‐cast NiTiSn, the average width of martensite twins in the NiTi lamellae exceeds 200 nm (Figure 2e). SAED pattern substantiates these are <011>_B19’_ type II twins (Figure 2f), which are commonly seen in coarse‐grained bulk martensitic NiTi.^[^ ^19^ ^]^ In this sense, it is the characteristic size of NiTi, namely lamellar thickness, that determines twinning morphology. Moreover, DSC curves and in situ high energy X‐ray diffraction (HE‐XRD) upon a thermal cycle exclude the existence of R‐phase in the as‐cast NiTiSn (Figure S9, Supporting Information).

To encapsulate, the as‐printed NiTiSn features a unique hierarchical structure induced by eutectic refinement. The first‐level architecture includes alternating NiTi and Ti_3_Sn nanolamellae. Within NiTi nanolamella, there exists the second‐level architecture composed of (001)_B19’_ compound nanotwins and R‐phase nanodomains.

### 2.2 Extraordinary Damping‐Elasticity Combination

**Figure 3:**
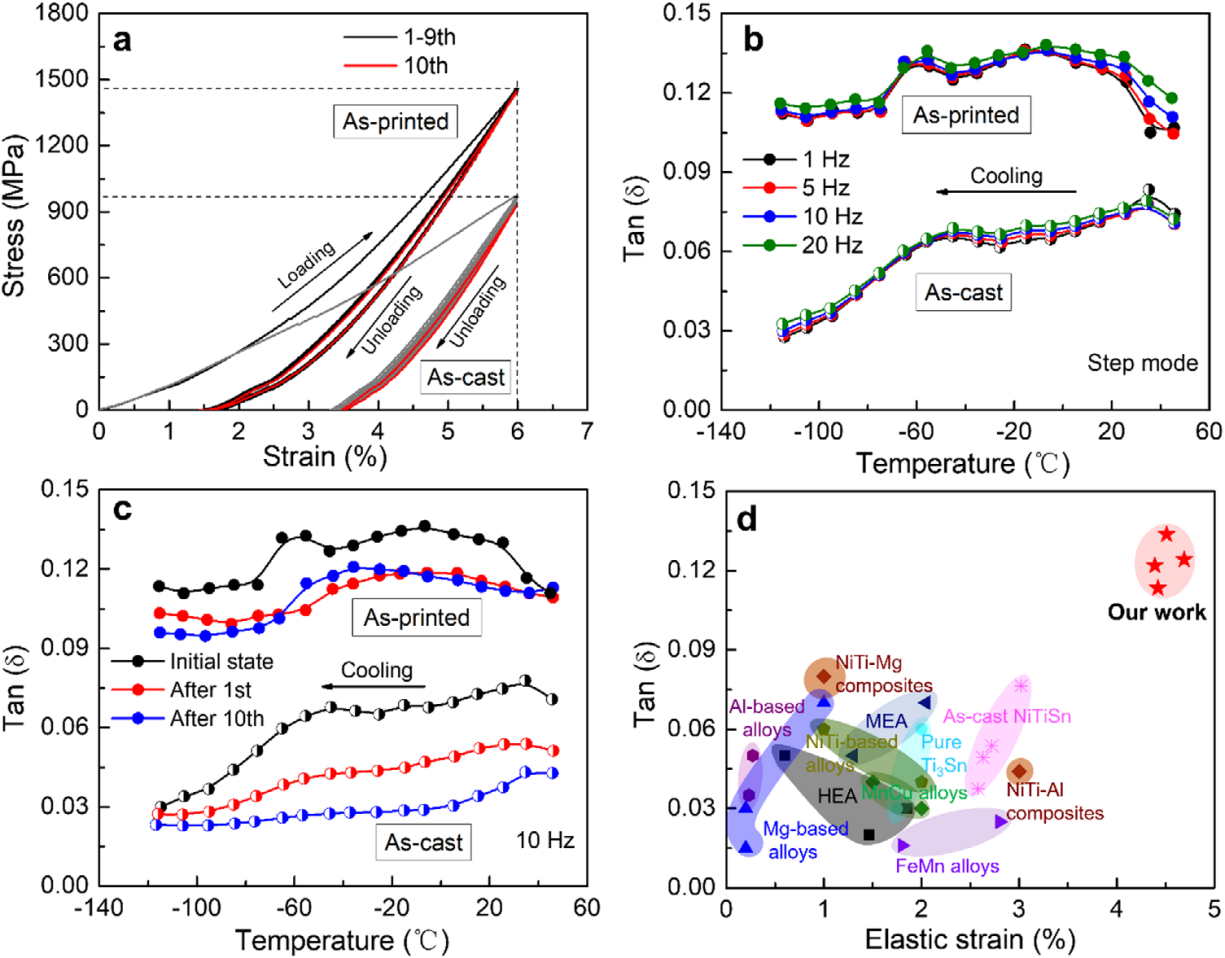
a) Compressive stress–strain curves of the as‐printed and the as‐cast NiTiSn samples during ten loading‐unloading cycles (peak strain of 6%) at room temperature; b) Temperature‐dependent damping capacity (evaluated by tanδ) of the as‐printed and the as‐cast NiTiSn under step mode (isothermal condition) at 1, 5, 10, and 20 Hz; c) Evolutions of tanδ (10 Hz) of the as‐printed and the as‐cast NiTiSn before and after mechanical cycling from a); d) Comparison of elastic strains and damping properties of our as‐printed NiTiSn, the as‐cast NiTiSn and other state‐of‐the‐art HDAs.^[^ ^7^, ^9^, ^17^, ^37^, ^38^, ^39^, ^40^, ^41^, ^42^, ^43^, ^44^, ^45^, ^46^ ^]^

The as‐printed NiTiSn is gifted with unparalleled mechanical performance. Regarding the compressive response under a certain peak strain, the as‐printed NiTiSn exhibits a larger elastic strain than the as‐cast NiTiSn at room temperature. Take 6% compression as an instance (**Figure** **3**a), the elastic strain of the as‐printed NiTiSn is 4.51% at the first cycle, superior to that of the as‐cast sample (2.70%). This represents a record‐high elastic strain limit among existing HDAs. During subsequent loading‐unloading cycles, the elastic strain and stress of the as‐printed sample are essentially constant, evidencing superior cyclic stability of the compressive behavior.

The as‐printed NiTiSn also exhibits excellent damping capacity (0.10 < tanδ < 0.14) under isothermal conditions over a wide temperature window (from 50 to −120 °C, Figure 3b), consistently outperforming the as‐cast NiTiSn (0.03 < tanδ < 0.08), the as‐printed binary NiTi (B19’) alloy (tanδ < 0.02, Figure S6, Supporting Information), and the most common commercial MnCu HDAs (tanδ = 0.04). ^[^ ^37^ ^]^ Figure 3c shows that during cyclic 6% compression, tanδ of the as‐printed NiTiSn stabilizes instantly at the first cycle, while tanδ of the as‐cast NiTiSn decreases continuously throughout repetitive overload. After 10 cycles, the tanδ of the as‐printed NiTiSn attenuates ≈15%, in sharp contrast to more than 50% drop of tanδ in the as‐cast NiTiSn, even though both samples show extremely low‐stress hysteresis in near‐linear elasticity that indicates nearly zero irreversible work upon cycling. To reiterate, the extraordinary damping functionality of the as‐printed NiTiSn has salient stability confronting cyclic overload.

A comparison of the damping capacity and the elastic strain of NiTiSn with other HDAs is displayed in Figure 3d. Clearly, 3D‐printed nanocomposite demonstrates a record‐breaking combination of high damping and large elastic strain surpassing state‐of‐the‐art HDAs.^[^ ^7^, ^9^, ^17^, ^37^, ^38^, ^39^, ^40^, ^41^, ^42^, ^43^, ^44^, ^45^, ^46^ ^]^ It means the proposed material design scheme is capable of overcoming the long‐standing damping‐elasticity paradox.

## 3 Discussion

### 3.1 Origin of Large Elasticity

**Figure 4:**
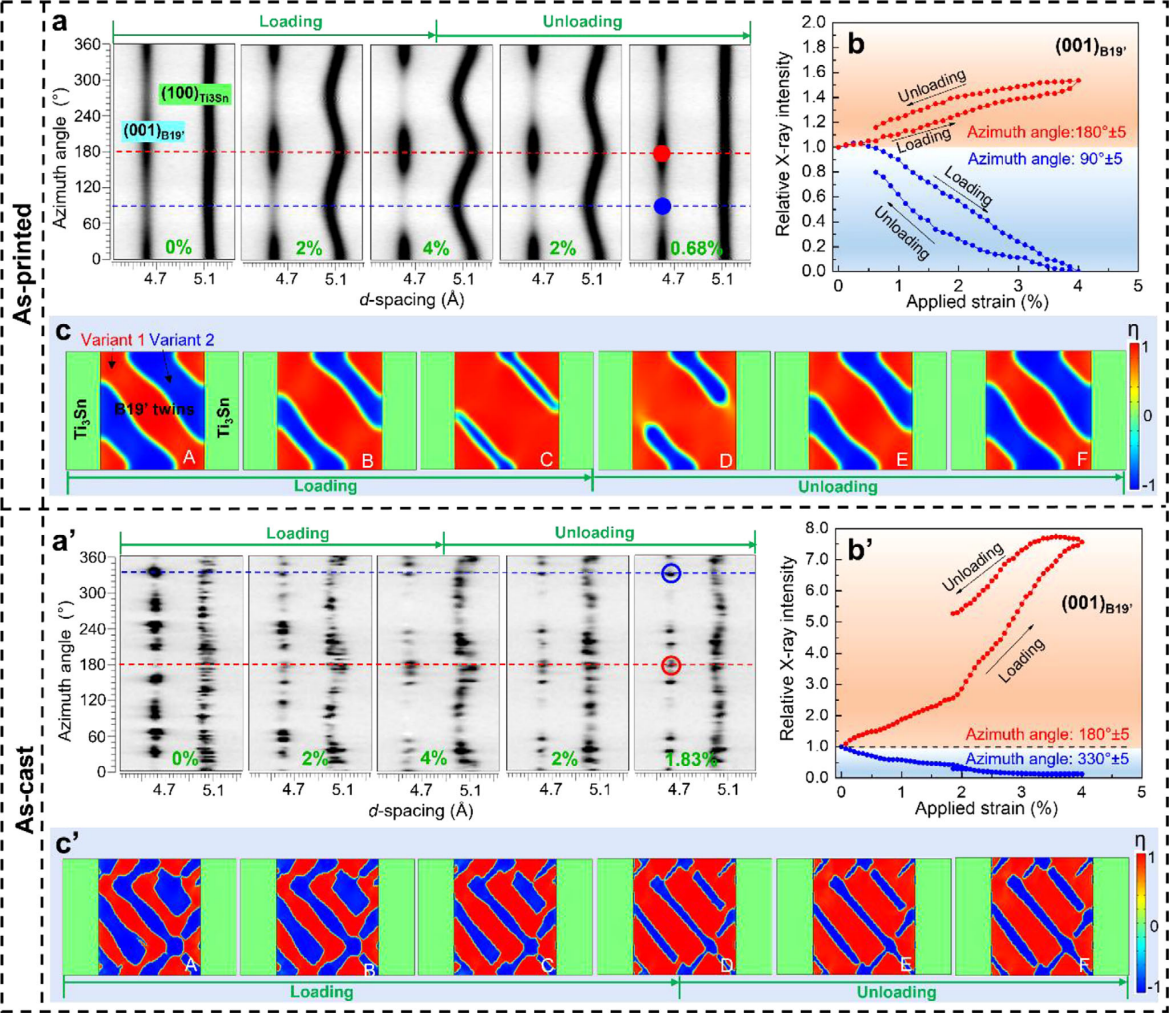
a‐a’) Evolution of 2D HE‐XRD patterns of (001)_B19'_ and (100)_Ti3Sn_ along the full Debye ring (azimuthal angle 0° to 360°) of the as‐printed (upper) and the as‐cast NiTiSn (lower) upon 4% compression at room temperature; b‐b’) Evolution of relative diffraction intensity of (001)_B19'_ along two azimuthal angles; c‐c’) Simulated evolution of order parameter (*η*) under different applied strains in calculated stress‐strain curves from phase field simulation of Figure S8 (Supporting Information). *η* = 1, −1 and 0 refer to variant 1 of B19’‐NiTi phase, variant 2 of B19’‐NiTi phase and Ti_3_Sn phase, respectively.

**Figure 5:**
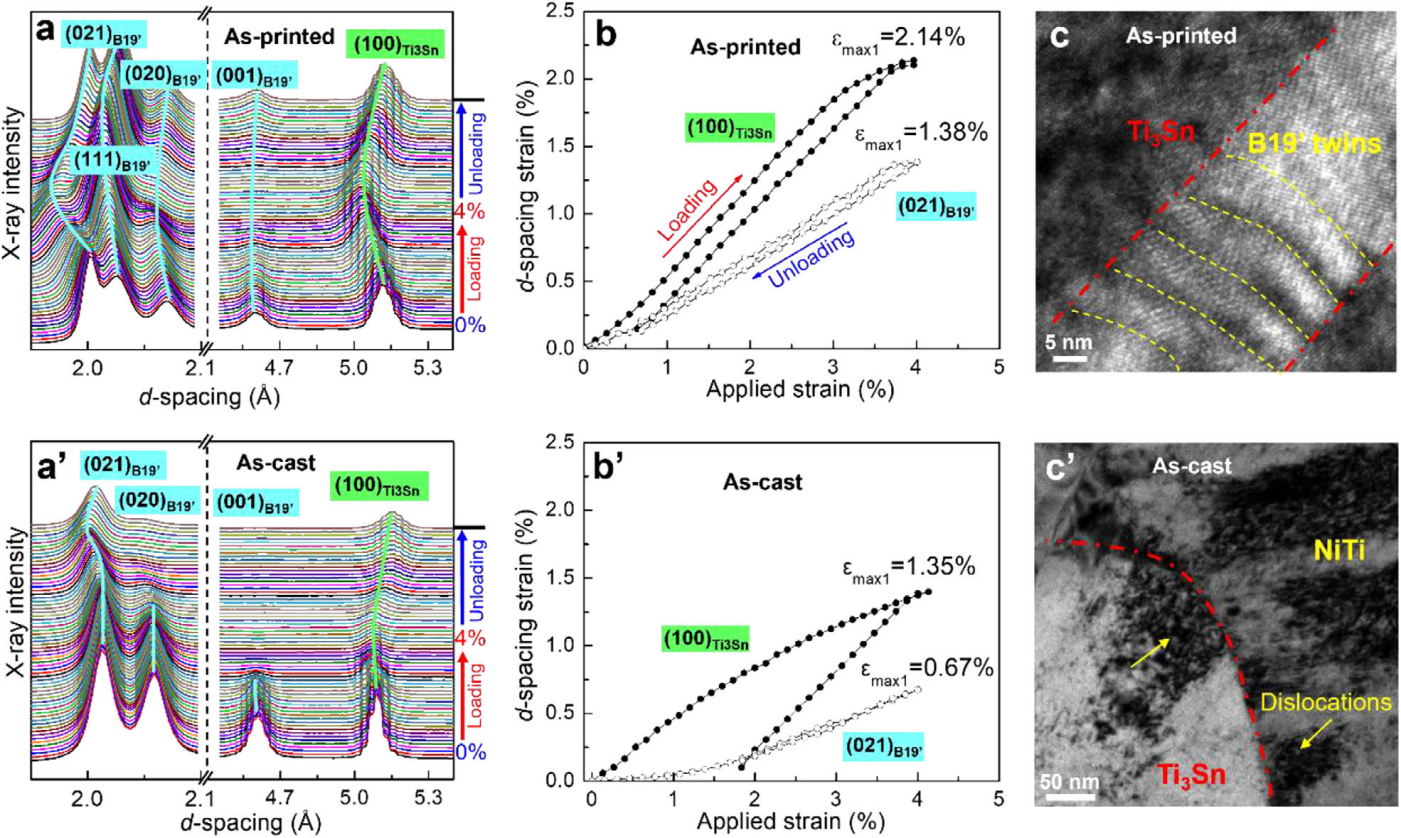
a‐a’) Evolution of 1D HE‐XRD patterns along the loading direction of the as‐printed (upper) and the as‐cast (lower) NiTiSn during 4% compression at room temperature; b‐b’) Evolutions of (100)_Ti3Sn_ and (021)_B19'_ lattice strains of the as‐printed and the as‐cast NiTiSn, respectively; c‐c’) TEM images of the as‐printed and the as‐cast NiTiSn after 4% compression. Lamellar boundaries are marked with red dash lines. For the as‐printed sample, dislocations are scarce, and twinning boundaries (marked by yellow dashed lines) are curved due to the internal stress after unloading. For the as‐cast sample, twinning structures disappear after unloading, and dislocation cells (pointed by yellow arrows) are generated near the lamellar interface.

To isolate the microstructural origin of large elastic strain in the as‐printed NiTiSn, in situ HE‐XRD tests were conducted during 4% compression at room temperature. In **Figure** **4**a, the diffraction profile of the as‐printed NiTiSn (extracted from 360°‐expanded 2D HE‐XRD patterns) reflects an unprecedented martensite reorientation mechanism caused by reversible stress‐induced detwinning‐twinning. Take (001)_B19'_ reflection as an instance, its intensity along the transverse direction (azimuthal angle of 180°) intensifies while that along the loading direction (azimuthal angle of 90°) decreases as applied strain increases (Figure 4b; Figure S7, Supporting Information). Therefore, (001)_B19'_ is an unfavored orientation in the compressive direction and B19’‐NiTi will detwinning during compression to form an energetically favored texture.^[^ ^19^ ^]^ After unloading, the diffraction profile tends to return to the initial distribution (Figure 4a), indicating that detwinned B19’‐NiTi twins again and the twinning substructure almost restores the initial state, in agreement with our phase field simulation (Figure 4c; Figure S8, Supporting Information) and TEM observation (**Figure** **5**c). To the best of our knowledge, this is the first report on reversible stress‐induced detwinning‐twinning in metallic materials. In comparison, (001)_B19'_ of as‐cast coarse grains has poor diffraction continuity (Figure 4a’) and still shows significant crystallographic preferred orientation, but the reversibility of twining behavior is very weak upon unloading (Figure 4b’,c’). This is also predicted by phase field simulation (Figure 4c’; Figure S8, Supporting Information) and cross checked by TEM (Figure 5c’). To this end, the reversible stress‐induced detwinning‐twinning plays a crucial role in the large elastic strain of the as‐printed NiTiSn (Figure 3a).

The internal biasing force exerted on the NiTi phase, which originates from the elastic constraint of the Ti_3_Sn phase, is transmitted more effectively as the lamellar thickness is reduced to tens of nanometers. The elastic energy stored by the detwinning process of the NiTi phase itself upon loading is also transformed into the re‐twinning driving force upon unloading.^[^ ^47^ ^]^ Moreover, because the as‐printed nanocomposite is less plasticized, the internal biasing stress imposed by the Ti_3_Sn phase is enhanced to restore the initial twinning morphology of the NiTi phase. Therefore, the strong interphase interaction in the first‐level architecture improves the reversibility of stress‐induced detwinning‐twinning.

Along with reversible detwinning‐twinning behavior, the large elastic strain of the as‐printed NiTiSn is attributed to the inhibition of plastic irreversibility. Derived from the 1D HE‐XRD pattern along the loading direction (Figure 5a,a’), the large hysteresis and significant residual of (100)_Ti3Sn_ lattice strain curve in Figure 5b’ indicate that the Ti_3_Sn phase in the as‐cast sample experiences notable plastic deformation after 4% compression, while the Ti_3_Sn phase of the as‐printed sample is deformed nearly elastically (Figure 5b). The maximum lattice strain of (100)_Ti3Sn_ in the as‐printed sample is up to 2.14%, much larger than that in the as‐cast sample (1.35%, Figure 5b’). In a similar way, so do (021)_B19’_ in addition to detwinning‐twinning deformation. TEM characterization demonstrates that massive dislocation cells are formed in both two phases near the lamellar interface in the as‐cast sample (Figure 5c’), but dislocations are rather scarce in the as‐printed sample (Figure 5c). The weakened dislocation slip propensity in the as‐printed NiTiSn is principally ascribed to lattice strain matching and lamellar boundary strengthening in the first‐level architecture.

### 3.2 Origin of Superb Damping

**Figure 6:**
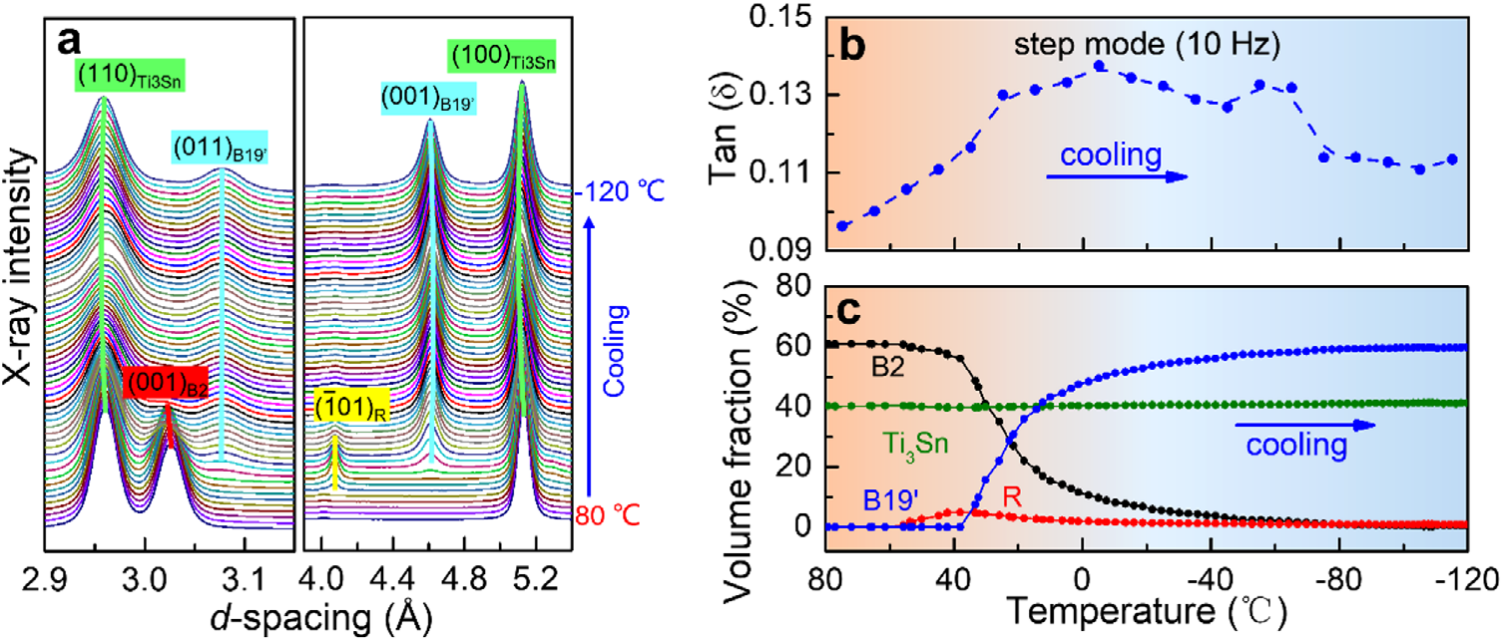
a) Evolution of fully integrated 1D HE‐XRD patterns upon cooling; b,c) Matchup of temperature‐dependent damping capacity (step mode at 10 Hz) and relative volume fraction of different phases upon cooling.

To uncover the underlying mechanisms responsible for the exceptional damping capacity in the as‐printed NiTiSn, in situ HE‐XRD was performed to record the phase evolution upon cooling (**Figure** **6**). Based on in situ HE‐XRD and TEM characterization, the improved damping capacity of the as‐printed NiTiSn in comparison to the as‐cast NiTiSn is attributable to two reasons. First, twinning morphology of B19’‐NiTi. It is well accepted that decreased shear strain eases the twinning process.^[^ ^18^, ^48^ ^]^ The shear strain of (001)_B19’_ compound twin (*s* = 0.238) is 15% smaller than that of <011>_B19’_ type II twin (*s* = 0.280),^[^ ^49^ ^]^ thus leading to higher mobility of twinning boundaries in the as‐printed NiTiSn. Besides, the twin width of the as‐printed NiTiSn is one to two orders lower than the as‐cast NiTiSn, generating a significantly higher density of twinning boundaries. These two factors are responsible for the intensive dissipation of B19’ nanotwins in the as‐printed NiTiSn. Second, the existence of R‐phase nanodomains. Its inherently highly movable interfaces will serve as complementary sources for energy dissipation ^[^ ^18^, ^50^ ^]^ in the as‐printed NiTiSn. In a nutshell, the second‐level architecture of the nanocomposite, namely (001)_B19’_ compound nanotwins, and R‐phase nanodomains within NiTi nanolamellae, is responsible for high damping capacity.

Additionally, nanolamellar boundaries impose severe mechanical resistance on the formation of B19’ martensite in NiTi nanolamellae. This not only promotes a little R phase (volume fraction less than 5% in Figure 6c) with a smaller scale to grow preferentially near the phase interfaces but also retards B19’‐related transformation to proceed progressively with continuously increased thermodynamic driving force upon cooling.^[^ ^18^, ^36^, ^50^ ^]^ When the temperature decreases, the as‐printed NiTiSn undergoes sustained, sluggish, and multiple transformations, including B2→R, B2→B19', and R→B19' (Figure 6a,c; Figure S9a, Supporting Information), in sharp contrast to the classical single B2→B19' transformation in the as‐cast NiTiSn (Figure S9b, Supporting Information). As a result of continuously increasing amount of nanotwinned B19', the dissipation capacity of the as‐printed NiTiSn exhibits an overall enhancement upon cooling until the martensitic transformation is completed ≈−60 °C, where the volume fraction of each phase has been constant and tanδ reaches the maximum value (Figure 6b). Moreover, the recovery of B19’ nanotwins after unloading (Figure 5c) effectively undermines the degradation of damping functionality during cyclic overload (Figure 3c). These aspects originate in wide‐temperature and overload‐tolerant superb damping capability in the as‐printed nanocomposite.

## 4 Conclusion

In summary, a NiTiSn nanocomposite with a unique two‐level hierarchical structure is manufactured via LPBF. The first‐level architecture including martensitic NiTi nanolamellae (≈57 nm) and reinforced Ti_3_Sn nanolamellae (≈40 nm) not only suppresses dislocation slip via lattice strain matching and lamellar boundary strengthening but also enables martensite reorientation through reversible stress‐induced detwinning‐twinning, all of which cooperate on superior elasticity. The second‐level architecture, i.e., (001)_B19’_ compound nanotwins and R‐phase nanodomains within NiTi nanolamellae, gives rise to sufficient internal friction for damping. As a consequence, the as‐printed nanocomposite exhibits unprecedented synergy of excellent damping capacity (tanδ > 0.10) and large elastic strain (more than 4.5%). The proposed material design strategy could be generally applied to other NiTi‐based eutectic systems (such as NiTi‐Nb, NiTi‐V, and NiTi‐Ti_5_Si_3_) to achieve multi‐functionality for special demands, thereby broadening their prospects as high‐performance functional and structural materials.

## 5 Experimental Section

### Material Fabrication

The pre‐alloyed Ti_58_Ni_34_Sn_8_ powder with a particle size of 15–60 µm was produced by gas atomization technology (Figure S1, Supporting Information). The as‐printed NiTiSn was prepared by an LPBF machine (M100‐T, Eplus, China) equipped with a 200 W Yb fiber laser beam with a diameter of 70 µm. A stripe rotation scanning strategy was deployed with a stripe width (laser scanning length) of 4 mm, a layer thickness of 40 µm, and a hatch rotation of 67° (Figure S2, Supporting Information). The as‐cast sample with the same element composition was produced from high‐purity elemental metals (purity > 99.8 wt.%) by vacuum induction melting.

### Microstructure Characterization

Microstructure observation was performed on a scanning electron microscope (SEM, Zeiss G310) and transmission electron microscopy (TEM, Tecnai G2 F20) at 200 kV equipped with a dispersive X‐ray spectroscopy (EDX) detector and Gatan double‐tilt holder. TEM samples were prepared by mechanical polishing followed by twin‐jet electro‐polishing in an electrolyte of 25 vol% HNO_3_ and 75 vol % CH_4_O at −30 °C and 30 V.

### Mechanical Tests

Damping tests were conducted using a dynamic mechanical analyzer (DMA, TA‐Q800,) under 3‐Point bending mode. The rectangular sample dimension was 4 × 2 × 30 mm^3^. The damping capacity (indexed by tanδ) was measured at a constant strain amplitude of 0.2%, a frequency range of 1–20 Hz, and temperatures from 50 to −120 °C. These were chosen to characterize intrinsic damping in line with common practices for HDA evaluation. Here the step mode (isothermal condition) was held for 5 min at each collected temperature to eliminate the interference of transient damping phenomena on the intrinsic damping magnitude of the sample.

Samples for compressive tests were cut into cylinders with a diameter of 3 mm and height of 6 mm by wire electric discharge. The compressive tests were carried out by a KQL universal testing machine at room temperature. The nominal strain rate was 5 × 10^−4^ s^−1^. The stress was equal to the applied force divided by the cross‐section area. An Epsilon mechanical extensometer was used to record the strain accurately. At least 3 samples were compressed under the same conditions to ensure the repeatability of the experimental data. No post‐processing heat treatment was applied to the LPBF‐fabricated samples prior to mechanical testing, maintaining strict comparability with the as‐cast reference material.

### In Situ High Energy XRD

HE‐XRD measurements were performed on the 11‐ID‐C beamline of the Advanced Photon Source at the Argonne National Laboratory, USA, using beam energy of 115 KeV (wavelength of 0.1173 Å) and beam size of 0.5 × 0.5 mm^2^. For phase analysis, full azimuth integration of the 2D HE‐XRD spectra was performed to get fully integrated 1D HE‐XRD spectra with software Fit 2D, where a CeO_2_ powder reference sample was used for calibration. The 1D HE‐XRD profile along the loading direction was integrated at the azimuth angle within 90 ± 5°. Diffraction profiles were fitted using the Gauss distribution function to determine the peak position (d‐spacing value). The d‐spacing strain was calculated as |d_hkl_ – d^0^_hkl_|/d^0^_hkl_ × 100%, where d^0^_hkl_ is the initial d‐spacing and d_hkl_ is the d‐spacing under thermomechanical loading.

### Phase Field Simulation

The lamellar structure of NiTiSn was simplified as a 2D sandwich with NiTi (B19’) phase in the middle (thickness of t_1_) and Ti_3_Sn phase on both sides (thicknesses of t_2_/2). According to the statistical results of Figs. 1 h and Figure S5 (Supporting Information), values of t_1_ = 60 nm and t_2_ = 40 nm were assigned to the as‐printed model, and t_1_ = 900 and t_2_ = 640 nm to the as‐cast model. Periodic boundary conditions were applied to all surfaces along a horizontal direction. As shown in Figure S8 (Supporting Information), a compressive displacement was imposed on the top surface along a vertical direction, and the vertical displacement of the bottom surface was fixed.

The total energy of the system consisted of Landau free energy, gradient energy, thermal energy, and elastic energy.^[^ ^51^ ^]^ As for the Ti_3_Sn phase, its elastoplastic deformation was modeled by a bilinear stress‐strain response. Its order parameter was assigned as η  =  0. Regarding the NiTi phase, two martensite variants were considered, which were sufficient to capture the key traits of martensite orientation in the 2D domain. The order parameter and the eigen strain tensor of Variant 1 were η  =  1 and $\varepsilon_{0}=\left(\begin{array}{cc} 0.05 & 0\\ 0 & 0.05 \end{array}\right)$, and those of Variant 2 were η  =   − 1 and $\varepsilon_{0}=\left(\begin{array}{cc} -0.05 & 0\\ 0 & 0.05 \end{array}\right)$. The temporal evolution of the order parameters was controlled by the time‐dependent Ginzburg‐Landau (TDGL) equations, which were solved by COMSOL Multiphysics.

## Conflict of Interest

The authors declare no conflict of interest.

## Supporting information



Supporting Information

## Data Availability

The data that support the findings of this study are available in the supplementary material of this article.
