# Supplementary material for: Overcoming the Damping–Elasticity Paradox via 3D‐Printed NiTiSn Nanocomposite
Source: Adv Sci (Weinh). 2025 Jun 10;12(33):e06410. doi: 10.1002/advs.202506410 (PMC12412537; doi:10.1002/advs.202506410)
Supplement: Supplementary file 1 — Supporting Information [file ADVS-12-e06410-s001.docx]

Supporting Information

Overcoming the Damping–Elasticity Paradox via 3D-Printed NiTiSn Nanocomposite

Bo Feng, Helong Liu, Hui Shen, Ying Yang*, Fangmin Guo, Lishan Cui, Yang Ren, Jie Chen, Shuke Huang, Yao Xiao*, Zhihui Zhang, Hongxiang Zong, Yinong Liu, Shijie Hao *


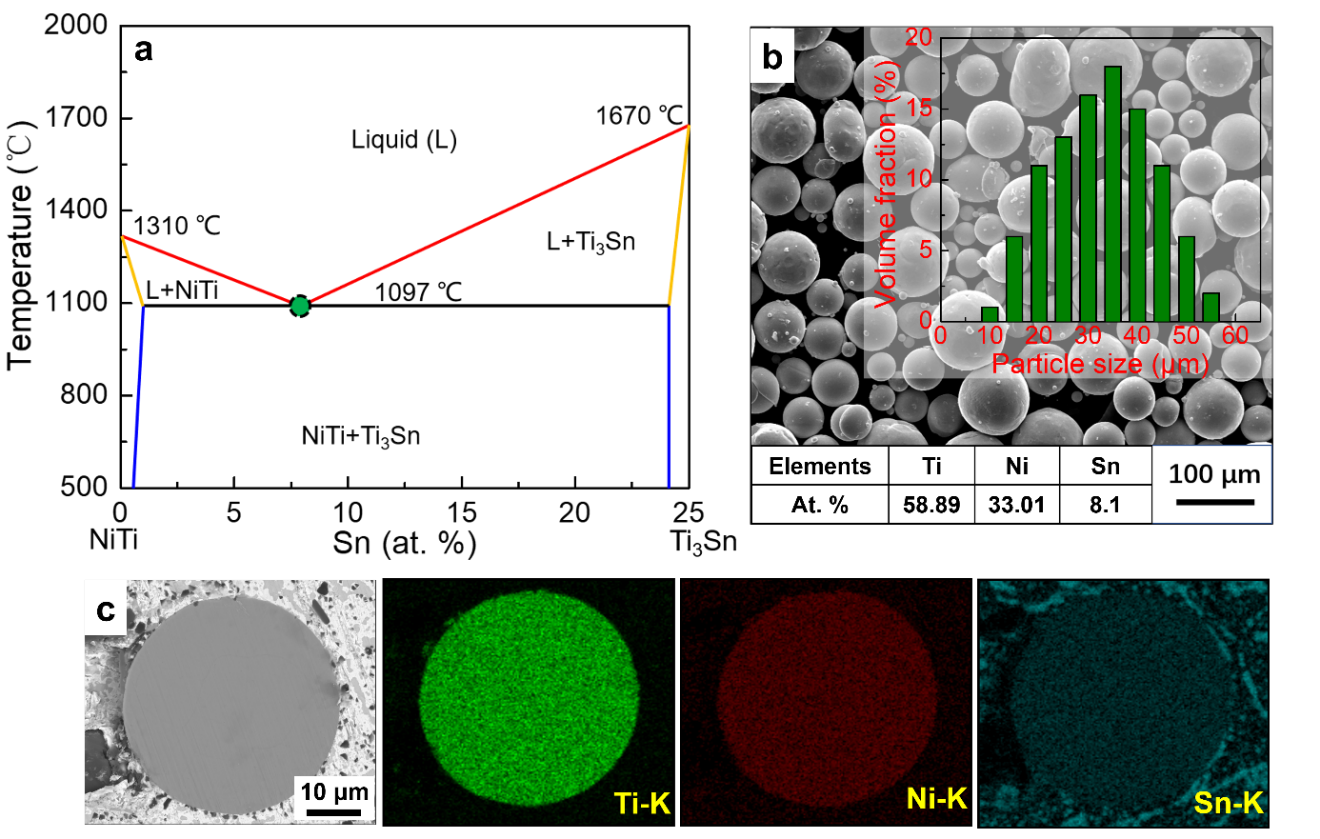


**Figure S1** a) The NiTi/Ti_3_Sn eutectic point (marked in green point) has a composition of Ni_34_Ti_58_Sn_8_ (at.%); b) Typical morphology and composition (the inset) of pre-alloyed NiTiSn powders with spherical particle size ranging from 10 to 55 μm. The oxygen content of the ingot before gas atomization is controlled below 100 ppm. c) STEM-EDX elemental maps of a pre-alloyed powder.


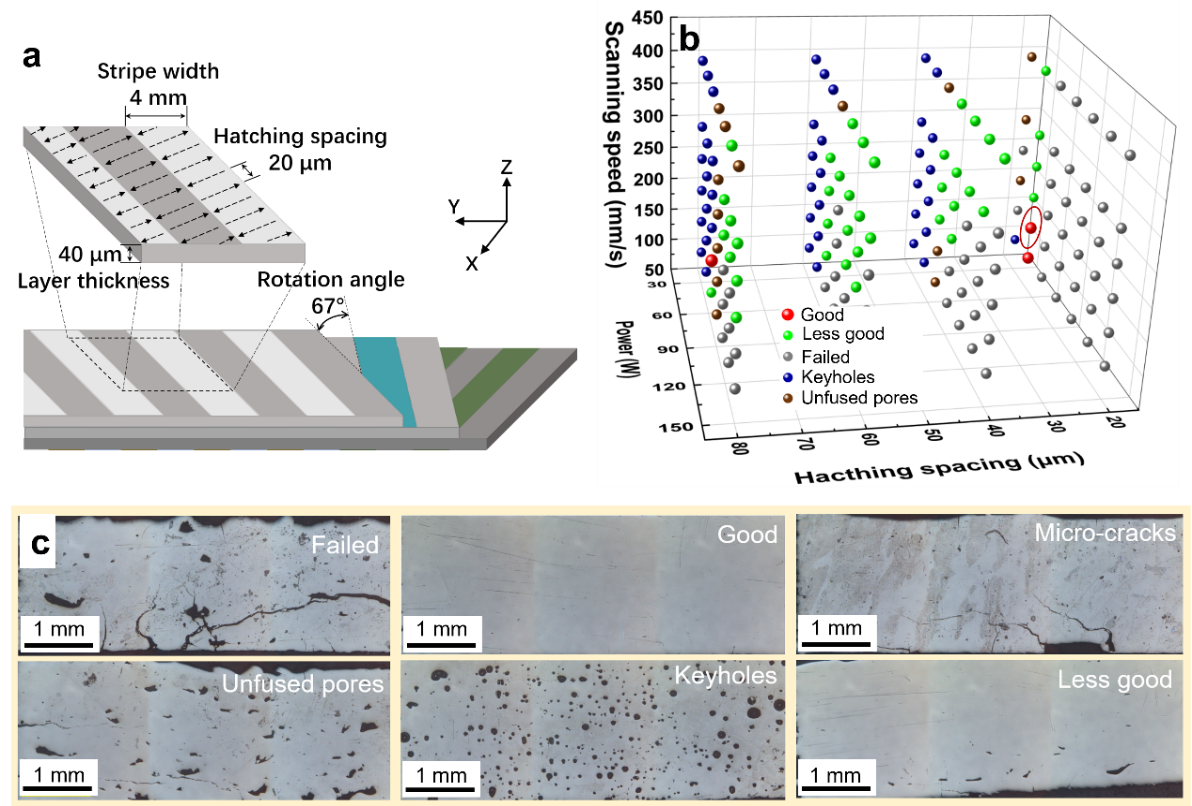


**Figure S2** a) Schematic diagram of the stripe rotation scanning strategy used for LPBF building process, The stripe rotation is 67º for successive layers; b) Formability map of LPBF-NiTiSn. The optimized LPBF parameter used in this study is highlighted in circle; c) Optical micrographs representing various forming status proposed in b). It is worth noting that sufficient interaction between molten pools and effective internal stress relief occur only at specific combinations of hatch spacing and scanning speed. The criterion for excellent formability requires further investigation.


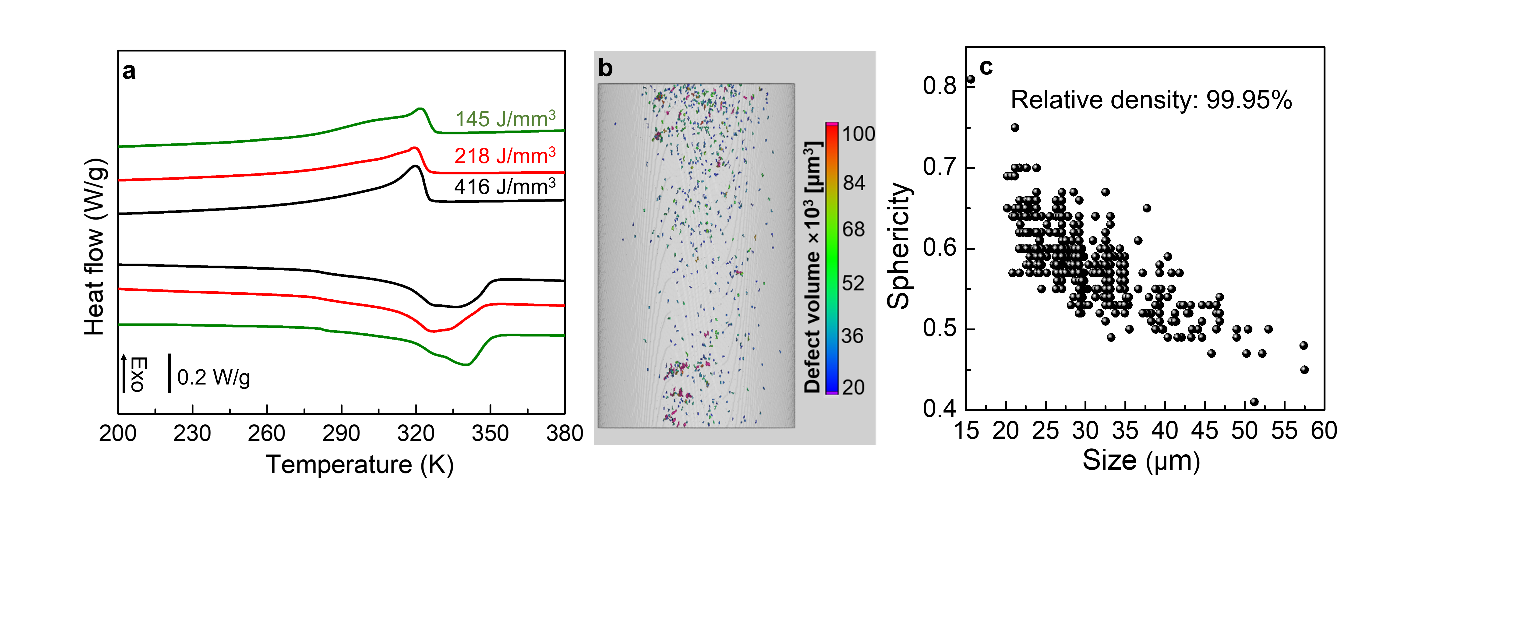


**Figure S3** a) DSC curves of NiTiSn samples fabricated under different volumetric energy densities; b) CT image of NiTiSn printed with the optimized parameter, which is called as-printed NiTiSn hereinafter and in the main text; c) Sphericities and diameters of pores in b).


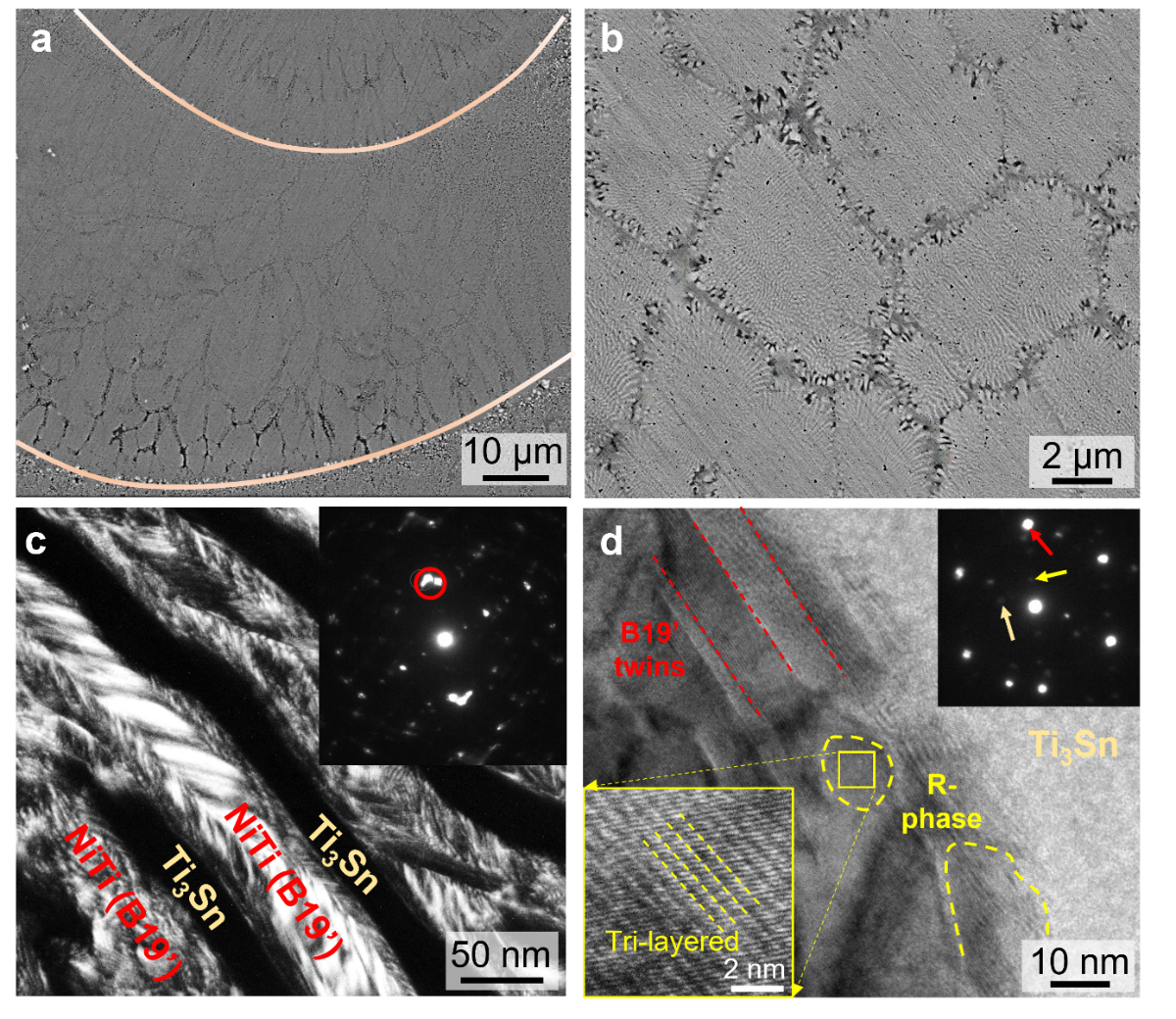


**Figure S4** Overview of the microstructure of the as-printed NiTiSn. a) Backscattered SEM image. The orange lines mark the near-hemi-spherical melt pool boundaries; b) An enlarged view of nearly equiaxed cellular structures inside the melt pools; c) Dark-field TEM image of nanolamellae taken by B19’-NiTi diffraction spots (encircled by red) in the inset. d) B19' nanotwins and R-phase nanodomains of the NiTi matrix at the NiTi/Ti_3_Sn nanolamellar boundaries. The tri-layered contrast of the atomic image and 1/3 diffraction positions evidence typical R-phase nanodomains.


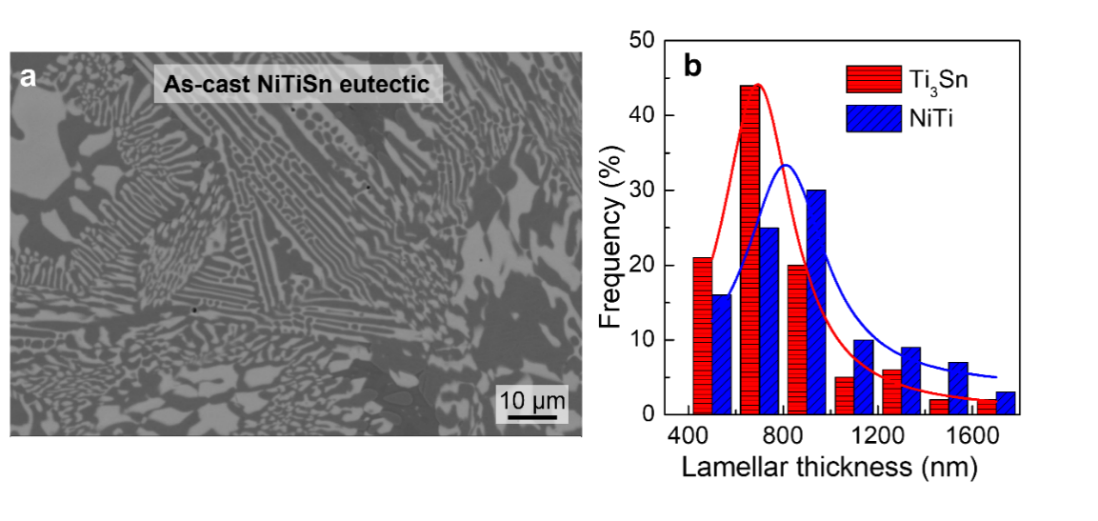


**Figure S5** a) Backscattered SEM image of the as-cast NiTiSn; b) Thickness distributions of lamellae in the as-cast NiTiSn.


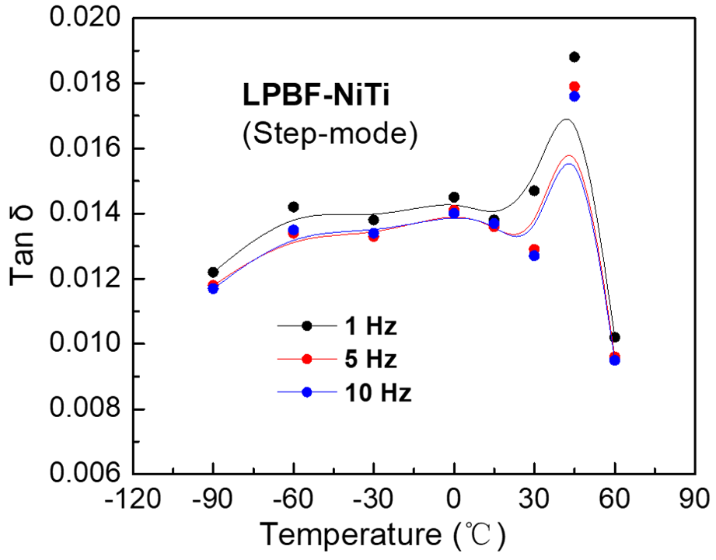


**Figure S6** Temperature-dependent damping capacity (evaluated by tanδ) of the equiatomic binary LPBF-NiTi under step mode (isothermal condition) at 1, 5, and 10 Hz.


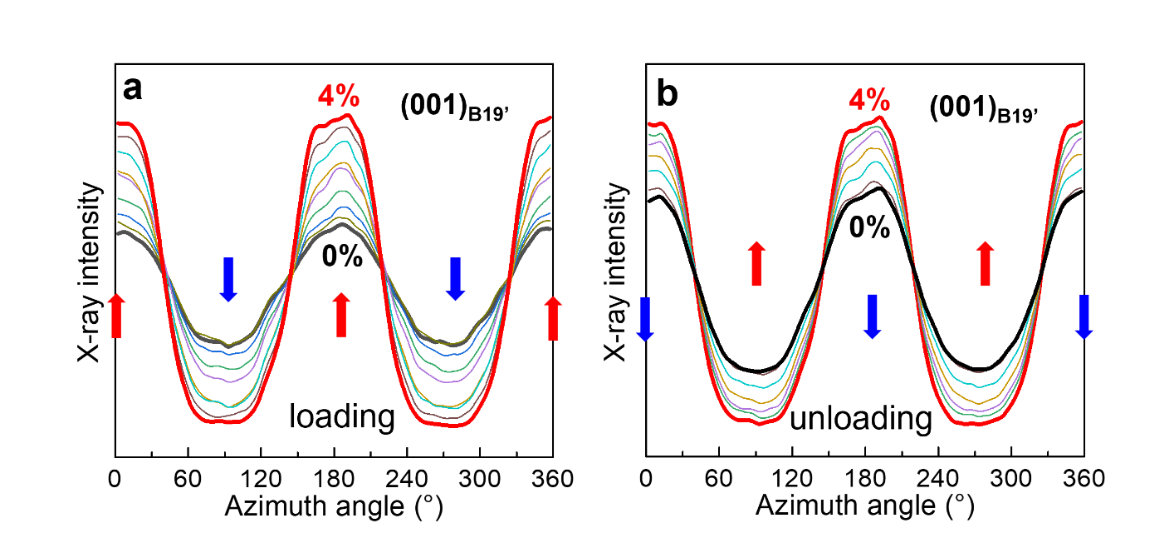


**Figure S7** Diffraction intensity of (001)_B19’_ along the full Debye circle (azimuth angle 0° to 360°) upon loading a) and unloading b) of 4% compression.


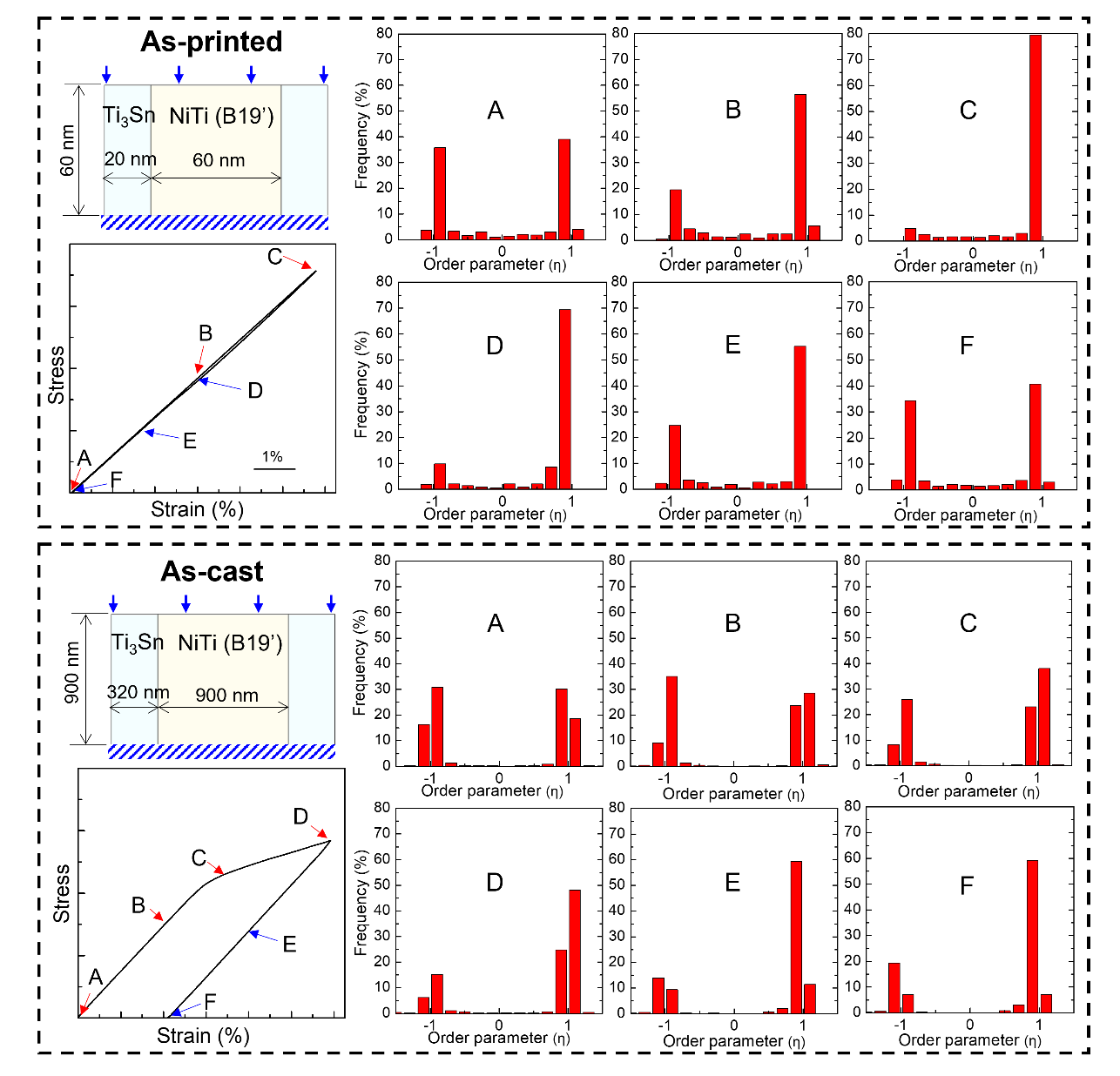


**Figure S8** The geometry of numerical sample used for phase field simulation and the histogram of order parameter in B19’-NiTi phase at representative strains in Figure 4.


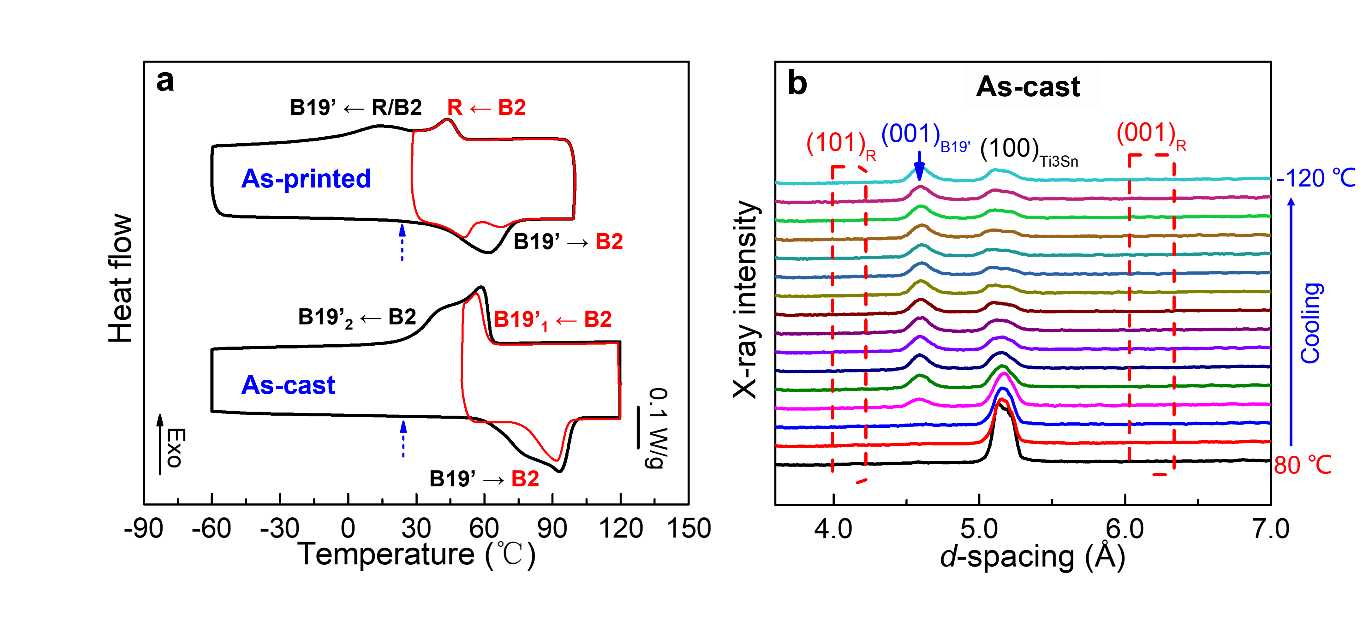


**Figure S9** a) DSC curves showing transformation temperatures of the as-printed and the as-cast samples. The blue dotted arrows are the state point of the sample (room temperature, cryogenic treatment state) for *in*-*situ* HE-XRD compression experiment in Figure 5. DSC curves from partial cool-heat cycle (red curves) indicate that the as-printed sample undergoes R-phase transformation (thermal hysteresis typically less than 10 ℃), while the as-cast sample undergoes a successive B2↔B19' martensitic transformation owing to the heterogeneous transformation by the eutectic lamellar interaction; b) Evolution of fully integrated 1D HE-XRD patterns of the as-cast NiTiSn during cooling. The red dashed box indexes the position where R-phase diffraction peaks should appear, thereby excluding the existence of R-phase in the as-cast NiTiSn.
